# Epidemiology of septic shock in prehospital medical services in five Colombian cities

**DOI:** 10.5935/0103-507X.20200006

**Published:** 2020

**Authors:** Diana Carolina López-Medina, Marcela Henao-Perez, Jaime Arenas-Andrade, Emel David Hinestroza-Marín, Fabián Alberto Jaimes-Barragán, Oscar Iván Quirós-Gómez

**Affiliations:** 1 Facultad de Medicina, Universidad Cooperativa de Colombia - Antioquia, Colombia.; 2 Departamento de Medicina Interna, Escuela de Medicina, Universidad de Antioquia - Antioquia, Colombia.; 3 División de Salud Pública, Facultad de Medicina, Universidad CES - Medellín, Antioquia, Colombia.

**Keywords:** Shock, septic/epidemiology, Sepsis, Emergency medical services, Home care services, Choque séptico/epidemiologia, Sepsis, Servicios médicos de urgencia, Servicios de atención de salud a domicilio

## Abstract

**Objective:**

To explore the association between demographic and clinical factors and the presentation of septic shock in patients treated by prehospital emergency services in five Colombian cities between 2015 and 2016.

**Methods:**

This was a cross-sectional study with retrospective data collection. Clinical and demographic data were collected from the medical records of patients diagnosed with sepsis who received prehospital care in five Colombian cities in 2015 and 2016. The diagnosis of septic shock was checked in 20% of the cases, generating two analyzed scenarios: observed and verified. Data were analyzed using the chi-square test, Student’s t test and an adjusted logistic regression model. Covariates with p < 0.05 were considered significant.

**Results:**

There was a higher frequency of septic shock in women (62.6%) and in individuals older than 80 years (64.5%), but these were not differentiating factors for septic shock. The most common source of infection was the urinary tract. In the observed scenario, age over 60 (prevalence ratio (PR): 3.22; 95% confidence interval (CI): 1.45 - 35.01) and history of cancer (PR: 1.20; 95%CI: 1.2 - 12.87) were the characteristics associated with septic shock, whereas in the verified scenario, chronic obstructive pulmonary disease (PR: 1.99; 95%CI: 1.26 - 7.14), history of cancer (PR: 1.15; 95%CI: 1.11 - 6.62) and presence of hypovolemia (PR: 1.41; 95%CI: 1.02 - 5.50) were observed.

**Conclusion:**

The most important risk factors for septic shock in prehospital care patients in five Colombian cities were oncological and pulmonary diseases and hypovolemia.

## Abstract (es)

**Objetivo:**

Explorar la asociación entre factores demográficos y clínicos con la presentación de shock séptico en pacientes atendidos en un servicio prehospitalario de emergencias en cinco ciudades colombianas entre los años 2015-2016.

**Métodos:**

Estudio de corte transversal con recolección retrospectiva de datos. Se recolectó información clínica y demográfica de las historias clínicas de pacientes con diagnóstico de sepsis que recibieron atención prehospitalaria en cinco ciudades colombianas en los años 2015 y 2016. Se realizó una verificación del diagnóstico de shock séptico en el 20% de los casos, dando origen a dos escenarios analizados: observado y verificado. Se analizó la asociación con pruebas de Chi cuadrado, t de Student y finalmente con un modelo de regresión logística ajustado. Se consideró covariables significativas aquellas con p < 0,05.

**Resultados:**

Se presentó una mayor frecuencia del evento en mujeres (62,6%) y en mayores de 80 años (64,5%), sin ser factores diferenciadores para shock séptico. El foco infeccioso más común fue urinario. En el escenario observado, los mayores de 60 años (RP: 3,22; IC95%: 1,45 - 35,01) y el antecedente de cáncer fueron las características asociadas con el shock séptico (RP: 1,20; IC95%: 1,2 - 12,87), mientras que en el grupo verificado fueron la enfermedad pulmonar obstructiva crónica (RP: 1,99; IC95%: 1,26 - 7,14), el antecedente de cáncer (RP: 1,15; IC95%: 1,11 - 6,62) y presencia de hipovolemia (RP: 1,41; IC95%: 1,02 - 5,50).

**Conclusión:**

Los factores de riesgo más importantes para shock séptico en pacientes de atención prehospitalaria en cinco ciudades colombianas fueron las enfermedades oncológicas, las pulmonares e hipovolemia.

## INTRODUCTION

Sepsis is a clinical syndrome of organ dysfunction resulting from an anomalous host response to infection, which can progress to a critical state called septic shock, characterized by microvascular endothelial dysfunction, dysregulated immune response and altered coagulation. Mortality in this state can reach up to 40%.^(1)^

In emergency services, the crude incidence rate of sepsis is 3.3 per 100 patients, surpassing those of acute myocardial infarction and stroke, with frequencies of presentation of 2.3 and 2.2 per 100 patients, respectively^(1)^. Specialized health centers have reported an increase in the frequency of septic shock, increasing from 2% in 1995 to 37% in 2006.^(2)^

Sepsis affects all age groups, with individuals at the extremes of life having greater susceptibility. In advanced age, several conditions are associated with septic shock, such as the presence of comorbidities, with cardiovascular and renal diseases recognized as risk factors for developing this critical state.^(3)^

Early identification of sepsis and septic shock is essential, and prehospital care has a special role in patient referral. It has been reported that up to 54% of hospital admissions for sepsis have been identified by outpatient services.^(4)^

In Colombia, the community-acquired sepsis rate in 2008 was 69%, and 16% of these individuals were in a critical state.^(5)^ The prevalence of sepsis and septic shock in outpatient medical services is unknown, as are the factors involved in the development of this critical state in the home. Therefore, the objective of this study was to explore the association between demographic and clinical factors and septic shock in patients treated in five Colombian cities at prehospital emergency services.

## METHODS

**Figure 1:**
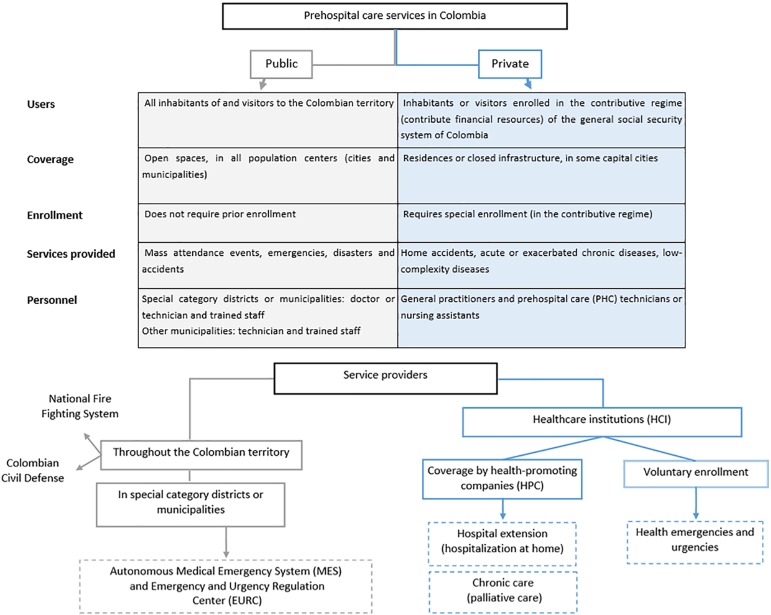
Flowchart of the structure of the Colombian Medical Emergency System.

This was a cross-sectional study with retrospective data collection from private, voluntary enrollment prehospital medical services with a varied number of users in Medellín (29,723), Cali (22,098), Bogotá (9,973), Barranquilla (4,546) and Cartagena (4,546) (Figure 1). We included 100% of the records of patients treated between January 1, 2015, and December 31, 2016, with a diagnostic impression of sepsis or septic shock, who were also older than 18 years. Records lacking data on the physiological variables necessary to establish shock conditions, such as blood pressure values, were excluded, as well as records in which drug-induced hypotension was established and those in which non-infectious shock was recorded.

**Figure 2:**
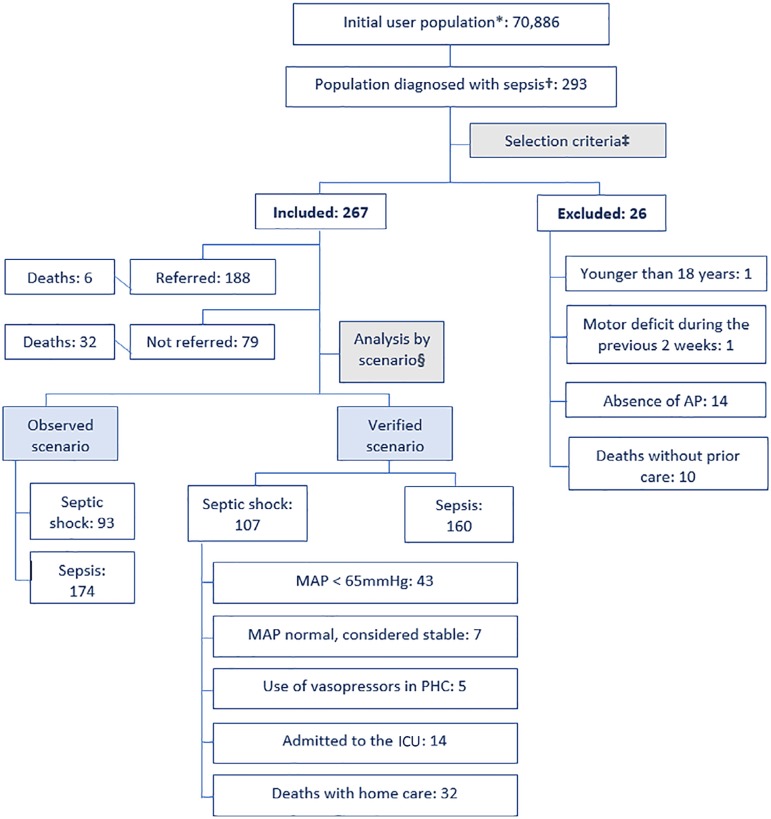
Flowchart of the application of the selection criteria. Source: created by the authors. *Population enrolled with the home emergency medical care company; †Population with septic shock or sepsis per ICD-10 code A.418 or A.419 for 2015 and 2016; ‡First verification; §Second verification. AP - arterial pressure; MAP - mean arterial pressure; PHC - prehospital care; ICU - intensive care unit.

Demographic and clinical data were collected through two verification steps of the event, a process that sought to confirm the infection, rule out another cause of hypotension and compare the outcome of patients who were or were not referred to hospitals. The first step was performed with 100% of the patients and involved reviewing the medical records up to two weeks before the diagnosis of sepsis in the home. The second step was performed with 100% of patients not referred to hospitals and involved tracking the outcome for two weeks after the diagnosis of sepsis. Of the referred patients, the event and its resolution were randomly assessed for 20% of patients by reviewing the hospital records, to then extrapolate the verified event to the remaining 80% of patients (Figure 2).

Demographic (sex and age) and clinical variables were collected, according to previous status (functional class and history of comorbidities and infections) and current status (vital signs and semiological findings). Mental state was assessed using the Glasgow Coma Scale, and states of mental confusion induced by hypovolemia or altered blood sugar levels were considered confounding factors.

The data were analyzed in SPSS 23, version 21 (CES University License). The chi-square and Student’s t tests were used according to the type of variable, and a final multiple logistic regression model was fitted to determine the association of demographic and clinical factors with septic shock developed in the home. These analyses were performed for two scenarios: the first corresponds to the observed scenario, based on the clinical judgment of the prehospital service physician, and the second corresponds to the verified scenario, with extrapolation of the confirmation of the event from hospital records.

This study was approved by the *Facultad de Medicina* of *Universidad CES*, under opinion #164 of the Operational Research Committee and #105 of the institutional Human Research Ethics Committee. Because it was a risk-free investigation in accordance with the provisions of Article 11 of Resolution 8430 of 1993 of the Ministry of Health, it was exempt from informed consent.

## RESULTS

Data were collected from a total of 267 medical records of patients treated in Medellín (n = 173), Cali (n = 65), Bogotá (n = 16), Barranquilla (n = 10) and Cartagena (n = 3) with a diagnosis of sepsis (ICD-10 codes A41.9 and A41.8). A total of 70.4% (n = 188) of the patients were referred to hospitals, of which the records of 38 patients were reviewed; of these patients, 65.8% (n = 25) required admission to the intensive care unit, with 36.8% (n = 14) presenting definitive septic shock, and 15.8% (n = 6) died in the hospital. Of the 79 patients not referred to a hospital, 40.5% (n = 32) died at home because of the infection.

The prevalence of septic shock was calculated in the verified scenario for each city, taking as the population the number of enrollees in each city. The highest prevalence of septic shock was found in Medellín (27 per 10,000 users). The prevalence ratio of septic shock/sepsis was highest in Bogotá, with 1.3 times more cases of septic shock than sepsis compared to the other cities (Table 1).

**Table 1:** Prevalence of septic shock and sepsis in prehospital medical services in Colombia by city and by observation period, in the verified scenario

	Septic shock	Proportion of septic shock (%)	Prevalence of septic shock*	Sepsis	Proportion of sepsis (%)	Prevalence of sepsis *	Septic shock/sepsis prevalence ratio
Medellín	79	73.83	26.58	94	58.75	31.63	0.84
Cali	15	14.02	6.79	50	31.25	22.63	0.30
Bogotá	9	8.41	9.02	7	4.38	7.02	1.29
Barranquilla	4	3.74	8.80	6	3.75	13.20	0.67
Cartagena	0	0.00	0.00	3	1.88	6.60	0.00

* Prevalence calculation: n/Number of enrollees. per 10,000 enrollees. Number of enrollees: Medellín (29,723), Cali (22,098), Bogotá (9,973), Barranquilla (4,546) and Cartagena (4,546).

### Analysis by scenario

In both scenarios, septic shock and sepsis occurred in greater proportions in women. The median age of the patients was 84 (interquartile range - IQR = 13) years. The recording of previous infections and functional class according to the Red Cross scale was low (48.7% and 48.3%, respectively), and these were not factors related to the event. Among previous infections, recurrent urinary tract infection was the most frequent in both scenarios, with a proportion of 35.5% (38/107). Consciousness, assessed according to alertness, orientation and responsiveness to the environment, was reported in 82% of patients, and capillary refilling was reported in 32%. Urinary infection was the most frequent source of infection among patients with septic shock and sepsis in both scenarios; the origin of the infection was not a differentiating factor for the development of the critical state in either scenario (Table 2).

**Table 2:** Demographic and clinical characteristics of patients with septic shock and sepsis in a prehospital medical care company in Colombia

Characteristics	Observed scenario					Verified scenario				
	Shock (93) n (%)	Sepsis (174) n (%)	PR	95%CI	p value	Shock (107) n (%)	Sepsis (160) n (%)	PR	95%CI	p value
Male	31 (33.3)	72 (41.4)	0.79	0.55 - 1.13	0.19	40 (37.4)	63 (39.4)	0.95	0.70 - 1.28	0.74
Age > 60 years	89 (95.7)	161 (92.5)	1.51	0.63 - 3.62	0.31	102 (95.3)	148 (92.5)	1.38	0.65 - 2.94	0.35
No history	4 (4.3)	2 (1.1)	-	-	-	5 (4.7)	1 (0.6)	-	-	-
One comorbidity	25 (26.9)	60 (34.5)	1	-	-	24 (22.4)	61 (38.1)	1	-	-
Two or more comorbidities	63 (67.7)	112 (64.4)	1.22	0.83 - 1.79	0.20	77 (82.8)	97 (55.7)	1.56	1.07 - 2.28	0.007║
Hypertension	58 (62.4)	105 (60.3)	1.13	0.79 - 1.63	0.74	67 (62.6)	96 (60)	1.17	0.84 - 1.62	0.66
Neurological sequelae*	47 (50.5)	94 (54)	0.95	0.68 - 1.34	0.60	54 (50.5)	87 (54.4)	0.96	0.71 - 1.31	0.53
Chronic obstructive pulmonary disease	23 (24.7)	40 (23)	1.10	0.75 - 1.61	0.74	34 (31.8)	29 (18.1)	1.58	1.17 - 2.14	0.01║
Diabetes	23 (24.7)	40 (28.7)	1.10	0.75 - 1.61	0.48	26 (24.3)	47 (29.4)	0.85	0.60 - 1.21	0.36
Cancer	29 (31.2)	23 (13.2)	1.95	1.41 - 2.7	< 0.001¶	28 (26.2)	24 (15)	1.69	1.28 - 2.23	0.02║
Chronic kidney disease	11 (11.8)	20 (11.5)	1.05	0.63 - 1.74	0.93	15 (14)	16 (10)	1.28	0.86 - 1.92	0.31
Missing data	1 (1.1)	1 (0.6)	-	-	-	1 (0.9)	1 (0.6)	-	-	-
Independent according to functional class†	6 (6.5)	5 (2.9)	1	-	-	7 (7.5)	7 (4.4)	1	-	-
Dependent according to functional class†	44 (47.3)	71 (40.8)	1.42	0.79 - 2.56	0.39	51 (54.8)	64 (40)	0.88	0.50 - 1.55	0.27
Missing data	43 (46.2)	95 (54.6)		49 (45.8)	89 (55.6)					
Altered consciousness	54 (58.1)	102 (58.6)	1.21	0.77 - 1.50	0.38	66 (61.7)	90 (56.3)	1.56	1.0 - 2.44	0.02║
Missing data	21 (22.6)	27 (15.5)	-	24 (22.4)	24 (15)	-				
Hypovolemia due to sensible losses‡	23 (24.7)	31 (17.8)	1.29	0.90 - 1.86	0.18	29 (27.1)	25 (15.6)	1.46	1.08 - 1.98	0.02║
Hypoglycemia§	2 (2.2)	0	2.93	2.47 - 3.47	0.11	2 (1.9)	0	2.55	2.18 - 2.97	0.15
Hyperglycemia§	3 (3.2)	8 (4.6)	0.79	0.29 - 2.12	0.75	4 (3.7)	7 (4.4)	0.93	0.42 - 2.07	1.00
Urinary tract infection	45 (48.4)	84 (48.3)	0.99	0.71 - 1.38	0.98	46 (43)	83 (51.9)	1.10	0.87 - 2.33	0.15
Pneumonia	32 (34.4)	66 (37.9)	0.89	0.63 - 1.27	0.56	42 (39.3)	56 (35)	0.83	0.82 - 1.49	0.48
Skin and soft tissues infection	7 (7.5)	21 (12.1)	0.69	0.35 - 1.34	0.24	10 (9.3)	18 (11.3)	0.92	0.54 - 1.57	0.61
Gastrointestinal tract infection§	5 (5.4)	9 (5.2)	1.02	0.49 - 2.10	1.00	7 (6.5)	7 (4.4)	1.26	0.73 - 2.17	0.44
Unidentified source	-	1 (0.6)	-	-	-	-	1 (0.6)	-	-	-

PR - prevalence rate; 95%CI - 95% confidence interval;

* Neurological sequelae due to cerebrovascular disease, head trauma or neurodegenerative disease;

† Functional class according to the Red Cross classification, Dependent: bedridden or chair-bound, needs help with activities of daily living and great difficulty ambulating (requires help from at least one person); Independent: difficulty ambulating and able to self-care;

‡ Hypovolemia: determined by clinical conditions such as sensible losses (vomiting and diarrhea);

§ Fisher’s exact test, between septic shock - sepsis and qualitative variable with expected values lower than 5.

║ p ≤ 0.05;

¶ p ≤ 0.01.

In total, 54.8% (51/107) of the patients with septic shock in the verified scenario needed help with all activities of daily living or were bedridden, but this was not a differentiating or associated factor (Table 2).

Some clinical parameters, such as vital signs (heart rate for the observed scenario; respiratory rate and oxygen saturation for the verified scenario) differed significantly between the groups (septic shock/sepsis) (Table 3). These differences between groups were not retained when adjusting for other covariates or confounding factors (Table 4).

**Table 3:** Vital signs of patients with septic shock and sepsis in prehospital medical services in Colombia

Vital sign	Observed scenario			Verified scenario		
	Shock (93)	Sepsis (174)	p value	Shock (107)	Sepsis (160)	p value
	Median (IQR)	Median (IQR)		Median (IQR)	Median (IQR)	
SBP (mmHg)	80 (30)	110 (40)	-	80 (30)	110 (30)	-
DBP (mmHg)	40 (11)	70 (20)	-	46 (16)	70 (20)	-
MAP (mmHg)	56.6 (13)	83.3 (20)	-	56.6 (18)	83.3 (23)	-
HR (bpm) *	100 (42)	109 (32)	0.02 *	105.5 (37)	104 (23.6)	0.524
RR (rpm) †	28 (12)	25 (12)	0.65	28 (17)	24 (12)	0.02‡
Oxygen saturation (%) †	91 (13)	92 (9)	0.79	90 (15)	93 (6)	0.002‡
T (degrees Celsius) *	37.5 (1.9)	38 (1.7)	0.06	37.9 (1.8)	38 (1.9)	0.66
Blood glucose (mg/dL) †	129 (62)	136.5 (55)	0.66	136 (66)	135 (54)	0.76

IQR - interquartile range; SBP - systolic blood pressure; DBP - diastolic blood pressure; MAP - mean arterial pressure; HR - heart rate; bpm - beats per minute; RR - respiratory rate; rpm - respirations per minute; T - body temperature.

* Kolmogorov-Smirnov test with normal distribution (p ≥ 0.05) for septic shock and sepsis in both scenarios and Student’s t test, between septic shock and quantitative variables with a normal distribution.

Observed scenario: septic shock: mean heart rate 94.8; (standard deviation 28.2), mean body temperature 37.4 (standard deviation 1.2); sepsis: mean heart rate 106.5 (standard deviation 22.6), mean body temperature 38 (standard deviation 1.2). Verified scenario: septic shock: mean heart rate 101.6 (SD 27.7), mean body temperature 37.7 (SD 1.2); sepsis: mean heart rate 103.2 (SD 23.6), mean body temperature 37.9 (SD 1.2).

† Mann-Whitney U Test;

‡ variables with statistacally significant association

**Table 4:** Demographic and clinical characteristics related to septic shock in patients treated by prehospital care services

Covariate (associated factors)	Observed scenario		Verified scenario	
	PR	95%CI	PR	95%CI
Risk age (≥ 60 years)	3.22	1.45 - 35.01‡	2.38	0.28 - 23.80
Sex (male)	0.99	0.37 - 2.61	1.14	0.71 - 2.98
History of COPD	1.08	0.58 - 3.41	1.99	1.26 - 7.14†
History of cancer (yes)	1.20	2.14 - 12.87‡	1.15	1.11 - 6.62†
Number of comorbidities (two or more comorbidities)	0.83	0.47 - 2.21	1.04	0.44 - 2.51
HR*	0.69	0.27 - 1.40	0.75	0.30 - 1.56
RR*	1.03	0.48 - 2.32	1.18	0.60 - 2.94
T*	0.82	0.88 - 1.28	0.87	0.38 - 1.66
Oxygen saturation*	1.01	0.47 - 2.21	1.49	0.97 - 4.51
Glasgow Coma Scale*	1.14	0.57 - 3.02	1.18	0.63 - 3.27
State of consciousness	1.03	0.43 - 2.75	1.10	0.58 - 3.61
Hypovolemia (sensible losses)	1.04	0.52 - 2.80	1.14	1.02 - 5.50†
Hypoglycemia (yes)	NC	NC	NC	NC
Hyperglycemia (yes)	0.97	0.09 - 3.63	1.00	0.18 - 4.81

PR - prevalence rate; 95%CI - 95% confidence interval; COPD - chronic obstructive pulmonary disease; HR - heart rate; RR - respiratory rate; T - body temperature; NC - not calculated due to low frequency.

* Recoded clinical signs, with normal values as a reference parameter and differences in the normal range considered altered.

Normal heart rate: 60 - 90 bpm. Normal respiratory rate: 12 - 22 rpm. Normal T: 36 - 38. Normal oxygen saturation: higher than 94%. Normal Glasgow Coma Scale score: >13.

† p ≤ 0.05,

‡ p ≤ 0.01.

Note: binary logistic regression analysis with Hosmer-Lemeshow goodness of fit test; explanatory capacity 16% and 18.2%, respectively, for the observed and verified scenarios. PR - prevalence ratio estimated from the odds ratio of the logistic regression; PR calculated by the conversion formula.^(6)^

The variables significantly associated with septic shock were different between scenarios:

For the *observed scenario*, age 60 years or older (prevalence ratio - PR 1.51; 95% confidence interval - 95%CI 0.63 - 3.62; bivariate analysis) (PR 3.22; 95% CI 1.45 - 35.01; multivariate analysis) and a history of cancer (PR 1.95; 95%CI 1.41 - 2.7; for the multivariate analysis) were the factors associated with the event in the correlation analysis (Tables 2 and 4, respectively).

For the *verified scenario*, a history of chronic obstructive pulmonary disease (COPD) (PR 1.58; 95%CI 1.17 - 2.14; bivariate analysis), (PR 1.99; 95%CI 1.26 - 7.14; multivariate analysis) and cancer (PR 1.69; 95%CI 1.28 - 2.23; bivariate analysis), (PR 1.15; 95%CI 1.11 - 6.62; multivariate analysis) were related to the event (Tables 2 and 4, respectively). A significant correlation was also found for hypovolemia (PR 1.46; 95%CI 1.08 - 1.98) (Table 2), and this factor was found to be associated with the event (PR 1.14; 95%CI 1.02 - 5.50) (Table 4).

The presence of two or more comorbidities was significantly related to septic shock (PR 1.56; 95%CI 1.07 - 2.28) in the bivariate analysis; however, statistical significance was lost in the adjusted analysis (Table 4).

## DISCUSSION

In this Colombian study of prehospital care services in five cities in the country, 15.1 cases of septic shock per 10,000 users treated were reported, with a greater frequency in elderly women and urinary tract infection as the main source. The most important associated factors were related to age, the presence of hypovolemia and previous comorbidities, mainly oncological or pulmonary diseases.

This study based the definition of septic shock on the pathophysiological concept of systemic hypoperfusion, using as a parameter a mean arterial pressure (MAP) of less than 65mmHg, adapted from the definition of the Third International Consensus for Sepsis. According to this definition, septic shock is identified when use of vasopressors is required for maintaining an MAP of at least 65mmHg and serum lactate level > 2 mmol/L (18mg/dL) despite adequate volume replacement.^(7)^ This conciliation was necessary because in prehospital medical care services in Colombia, there is an absence of tools to determine the serum lactate level, in addition to low adherence to protocols due mainly to the scarcity of resources and the relative closeness to high-complexity health services.

The estimated incidence of sepsis according to the World Health Organization among patients hospitalized in the United States in 2008 was 32 out of 10,000 patients.^(8)^ The findings of the present study approach these values, and they would exceed them if considering the prevalence of the cities with higher frequencies (Medellín, Cali and Bogotá); in this case, the prevalence of septic shock would increase to 42.3 per 10,000 users treated.

Regarding demographic factors, the finding for sex was consistent with a prospective multicenter study conducted in Colombia, where 52.3% of patients with sepsis were women,^(5)^ but different from that reported in other geographic areas, where this phenomenon occurs predominantly in men.^(9-11)^

Patients older than 80 years were the most affected, which can be explained by the fact that the proportion of elderly individuals in the population is increasing, and as is known, extreme age predisposes patients to the development of sepsis when faced with an infection.^(12)^ In a previous study, the incidence of sepsis in people over 85 years of age was 26.2 cases per 1,000 inhabitants globally, which is 100 times higher than that observed in people between 5 and 14 years of age.^(12)^ Variables that may favor the presentation of sepsis in the elderly population include frequent comorbidities, long-term institutionalization, decreased functional status, impaired immune function and the growing demand for hospital health services.^(13)^

Because older adults tend to have one or more of the predispositions mentioned above, this study reported age greater than 60 years as a cofactor associated with septic shock when adjusted for the other covariates in the observed scenario.

Although its statistical significance was not retained, the presence of two or more comorbidities was found in the bivariate analysis to be a factor related to septic shock (PR: 1.56; 95%CI 1.07 - 2.28), with a significant difference for the verified scenario. These estimates have been reported to be even higher in other studies, where it was described that the presence of comorbidities increases the risk of sepsis and septic shock six times and the mortality rate at 30 days up to 22 times.^(14)^ The loss of statistical significance could be explained by the sample size or by the number of variables introduced in the model.

In this study, arterial hypertension was the most frequent comorbidity, followed by neurological sequelae. However, COPD was the pathology most associated with the development of septic shock in the verified scenario, and the presence of cancer was the pathology most associated with the development of septic shock in the observed and verified scenarios. COPD has been reported in some studies as a factor related to septic shock.^(15)^ In other studies conducted in Colombia, diabetes is the most predominant pathology in patients with sepsis, followed by COPD and chronic renal failure (19%, 12% and 11%, respectively), with COPD being a differentiator between having an infection with sepsis, infection without sepsis or no infection.^(16)^

Regarding cancer as an antecedent related to septic shock, it has been described that quantitative and functional defects in immune system cells, in addition to defects caused by cytotoxic chemotherapy, make patients with cancer more susceptible to bacterial or fungal infections that trigger severe sepsis.^(17)^ In the Netherlands, Van der Wekken et al. found that cancer is the most prevalent comorbidity in patients with sepsis,^(18)^ and in Korea, Park et al. reported it as the third most common comorbidity, representing a significantly greater risk for men.^(19)^ However, in Spain, Romero et al. found no significant difference between critically or semi-critically ill patients and stable patients in regard to the type of comorbidity.^(20)^

Among the clinical macrovascular signs of systemic hypoperfusion is hypotension (MAP < 70mmHg), which has been considered since the late nineteenth century as the main diagnostic tool to define shock.^(21)^ Tachycardia and altered state of consciousness are also accepted, among others. Because MAP is the coefficient of an equation involving systolic blood pressure (SBP) and diastolic blood pressure (DBP), the clinical dependence of these factors explains the strong association of SBP and DBP with septic shock found in this study. In addition, the use of MAP as the classification criterion in this study precluded the inclusion of SBP and DBP in the multivariate correlation analysis.

Heart rate was a differentiating factor for septic shock in the observed scenario, as also described in a study conducted in Denver, Colorado, which allowed developing a Sepsis Alert Protocol for prehospital emergency care personnel.^(15)^ State of consciousness followed the same pattern in the verified scenario, despite having 38% underreporting, explaining the loss of statistical significance in the multivariate analysis and reflecting the poor recognition of this sign as linked to the event of interest, a problem not unknown in other countries. For example, in Germany, Metelmann et al.^(22)^ recommend improving records for the timely recognition and implementation of algorithms for the treatment of septic shock.^(23)^

Respiratory rate in this study showed great variability; more than half of the individuals had altered values (> 22rpm) in both patients with and without shock, a condition that, at the end of the analysis, did not represent a variable associated with septic shock in the verified scenario, a finding that contrasts with that reported by Baez et al., who identified it as responsible for admission to the intensive care unit.^(24)^

Oxygen saturation was reported in 99.2% of patients, which in the multivariate analysis was not a factor associated with septic shock, unlike what has been reported in other studies, in which it is described as a risk factor.^(25)^

Body temperature in some studies is recognized as an independent factor, with variations of 1ºC higher in patients with sepsis than in those without sepsis.^(26)^ This study found no significant differences in this variable.

State of consciousness is recognized as one of the main altered clinical signs during septic encephalopathy. This sign can have different degrees, and its origin can be multifactorial, among which electrolyte imbalance, changes in blood volume, hypoglycemia, hepatic or renal dysfunction, bacterial endotoxins and other products of pathogenic agents are recognized.^(27)^ This is why our study adjusted the multivariable model by the presence of confounders for altered mental state, such as hypovolemia, hypoglycemia and hyperglycemia.

The most common source of infection reported in this cohort of patients with septic shock was urinary, similar to that reported for Colombia in 2011 by Rodríguez et al., where the most frequent diagnosis was urinary tract infection (28.6%),^(5)^ similar to the study by Caraballo et al., in which the urinary tract was the most common source of infection in 27.8% of cases.^(28)^ These results are contrary to what was described in an international study of the prevalence and outcomes of infection in intensive care units (EPIC II), where the main source of infection leading to sepsis was the lungs (64% of cases), followed by the abdomen (20%), bloodstream (15%) and renal and genitourinary tracts (14%).^(29)^ Similar values were observed in studies focused on prehospital settings, where the most frequent source of infection was the respiratory tract followed by the urinary tract.^(30,31)^

Despite the strategies used to collect information, in this study, a significant percentage of missing data was found in regard to history of infections (41%) and clinical conditions, such as the functional class of the patient (55%) and capillary refilling (68%). In a study by Matthaeus-Kraemer et al., a lack of information in medical records that leads to a delay in the detection and early treatment of severe sepsis and septic shock was considered a barrier in prehospital care.^(32)^

In addition to correcting underreporting, changes in paradigms are also required for all actors of the health system in Colombia,^(33)^ as well as the implementation of strategies that allow the identification of sepsis in early stages and therefore the early management, referral and admission of these patients because, as has been reported, the probability of death increases by 20% for each hour the initiation of antibiotics is delayed once the patient enters the hospital.^(34,35)^

## CONCLUSION

Septic shock is a frequent event in patients treated via prehospital services in Colombia, with a similar frequency between men and women but with a greater proportion in older adults. The factors that were found to be associated with septic shock, which should call the attention of physicians treating this population, were chronic obstructive pulmonary disease, cancer and hypovolemia in the verified scenario; for the observed scenario, the factors were age older than 60 years and history of cancer.

Better standardization in the collection of relevant information for diagnosis is required, which leads to timely detection during prehospital care and referral to a hospital for management and control.
